# Effect of a High-Intensity Tandem Bicycle Exercise Program on Clinical Severity, Functional Magnetic Resonance Imaging, and Plasma Biomarkers in Parkinson's Disease

**DOI:** 10.3389/fneur.2020.00656

**Published:** 2020-07-24

**Authors:** Carolina Segura, Mauricio Eraso, Javier Bonilla, Carlos O. Mendivil, Giselle Santiago, Nicolás Useche, Oscar Bernal-Pacheco, Guillermo Monsalve, Laura Sanchez, Enrique Hernández, Maria José Peláez-Jaramillo, Allison Cárdenas-Mojica

**Affiliations:** ^1^Vida Activa, Department of Internal Medicine, Fundación Santa Fe de Bogotá, Bogota, Colombia; ^2^School of Medicine and Health Sciences, Universidad del Rosario, Bogota, Colombia; ^3^School of Medicine, Universidad de los Andes, Bogota, Colombia; ^4^Endocrinology Section, Department of Internal Medicine, Fundación Santa Fe de Bogotá, Bogota, Colombia; ^5^Radiology and Diagnostic Imaging Department, Fundación Santa Fe de Bogotá, Bogota, Colombia; ^6^Neurology Department, Fundación Santa Fe de Bogotá, Bogota, Colombia; ^7^Neurosurgery Section, Department of Surgery, Fundación Santa Fe de Bogotá, Bogota, Colombia

**Keywords:** Parkinson disease, exercise, tandem bicycle, magnetic resonance imaging, biomarkers

## Abstract

**Rationale:** The optimal modality, intensity, duration, frequency, and dose–response of exercise as a therapy for Parkinson's Disease (PD) are insufficiently understood.

**Objective:** To assess the impact of a high-intensity tandem bicycle program on clinical severity, biomarkers, and functional MRI (fMRI) in PD.

**Methods:** A single-center, parallel-group clinical trial was conducted. Thirteen PD patients aged 65 or younger were divided in two groups: a control group and an intervention group that incorporated a cycling program at 80% of each individual's maximum heart rate (HR) (≥80 rpm), three times a week, for 16 weeks. Both groups continued their conventional medications for PD. At baseline and at the end of follow-up, we determined in all participants the Unified Parkinson's Disease Rating Scale, anthropometry, VO_2_max, PD biomarkers, and fMRI.

**Results:** VO_2_max improved in the intervention group (IG) (+5.7 ml/kg/min), while it slightly deteriorated in the control group (CG) (−1.6 ml/kg/min) (*p* = 0.041). Mean Unified Parkinson's Disease Rating Scale (UPDRS) went down by 5.7 points in the IG and showed a small 0.9-point increase in the CG (*p* = 0.11). fMRI showed activation of the right fusiform gyrus during the motor task and functional connectivity between the cingulum and areas of the frontal cortex, and between the cerebellar vermis and the thalamus and posterior temporal gyrus. Plasma brain-derived neurotrophic factor (BDNF) levels increased more than 10-fold in the IG and decreased in the CG (*p* = 0.028). Larger increases in plasma BDNF correlated with greater decreases in UPDRS (*r* = −0.58, *p* = 0.04).

**Conclusions:** Our findings suggest that high-intensity tandem bicycle improves motor function and biochemical and functional neuroimaging variables in PD patients.

**Trial registration number:** ISRCTN 13047118, Registered on February 8, 2018.

## Introduction

Parkinson's disease (PD) has a prevalence rate of 1–2 per 1,000 of the population worldwide (1). Despite L-DOPA treatment and optimal medication, around 36–50% of patients with PD will present motor complications such as dyskinesia and motor fluctuations (2, 3), and 78% will eventually develop dementia (4), leading to various degrees of disability and reduced quality of life. In working-age adults, PD also affects their economic productivity (5). All these issues have motivated an intense search for effective adjuvant therapies to complement current medications and improve management of PD. Exercise has been proven to improve motor function and mobility (6, 7) and enhance clinical functions including strength, gait, and balance (8). It also has a positive impact on cognitive function (9), patient-reported quality of life, and mental health (7, 10). The postulated mechanisms reported include enhanced cerebral oxygenation (11), improved plasticity of cortical striatum (12), release of humoral factors and neurotransmitters (7, 13, 14), and stimulation of dopaminergic neurons still functioning (15). More specific studies suggested that high-speed, complex goal-directed exercise such as cycling (high velocity, complexity, and repetition) can induce activity-dependent neuroplasticity (6, 16). High-cadence tandem cycling has shown to reduce PD symptoms in both upper and lower extremities and to increase brain activation as measured by functional MRI (fMRI) (6, 17–19).

A tandem bicycle has a drive train that mechanically links the pedals through a timing chain. When one of the riders is a PD patient, the higher cadence and consistency in pedaling that a healthy partner may deliver aids to achieve and maintain a pedaling rate that is greater than the former's preferred voluntary rate. Forcing the pace facilitates mechanically augmented moderate- and high-intensity aerobic exercise and provides sensory motor input possibly impacting motor speed and control via neurophysiological mechanisms. Some literature has shown that forced (tandem) cycling at a high cadence improves motor function and promotes functional improvement in PD. However, the mechanisms that underline those benefits are still being debated (6).

Low-intensity progressive cycling training can improve motor function in PD, especially akinesia (20).

However, the mechanisms underlying the improvement of motor function in PD patients after cycling are not known.

Several blood-borne molecules have been involved in mediating the effects of exercise on PD. One of them is brain-derived neurotrophic factor (BDNF), a protein with a potent effect on dopaminergic neuron survival and morphology (21, 22). Other potential mediators include regulated on activation, normal T expressed and secreted (RANTES), a chemokine correlated with motor involvement in PD patients (23); cathepsin D, a protease whose activity has been related to cell death in primate models of PD (24); myeloperoxidase (MPO), an enzyme with neurotoxic effects in a rodent model of PD (25); neural cell-adhesion molecule (NCAM) (26), a potential neurotrophic mediator; and platelet-derived growth factor isotype BB (PDGF-BB), whose regenerative properties have been demonstrated in a rat model of PD (27).

These mechanisms of action of exercise at the molecular and cellular level should also be reflected by changes in patterns of brain activation in functional neuroimaging studies. fMRI has revealed signature alterations in PD, including hyperactivation of cerebellar (28, 29) and primary motor cortex (30, 31). Recently, accelerated loss of hippocampal volume (32), gray matter atrophy of posterior cingulate cortex (33), and reduced connectivity in the prefrontal-limbic network (34) have also been linked to mild cognitive impairment and depressive symptoms in PD. Some studies have shown increased exercise-induced activity in the ventral striatum and increased repetitive transcranial magnetic stimulation-evoked dopamine release in the caudate nucleus (17). Nonetheless, the influence of highly challenging exercise therapy on fMRI features of PD is insufficiently known.

Despite abundant evidence addressing the clinical impact of exercise on PD, the optimal modality, frequency, and intensity of exercise practice as an adjuvant therapy in PD have not been established. The same can be said about the mechanistic pathways that result in the reported improvements.

Some of the heterogeneity of results regarding benefits of exercise in PD may arise from the fact that voluntary and self-paced exercise elicit distinct neural responses and may yield different outcomes (35). Animal and human trials with forced exercise (i.e., practiced above the intensity preferred by the subject) have shown positive results (36, 37). Furthermore, a prior trial proved a tandem bicycle intervention to be feasible in PD and to improve general physical performance measures (37).

With this context, we aimed to assess the impact of a high-intensity exercise program based on tandem bicycle training and on diverse aspects of PD including standardized scales of clinical symptoms, biochemical markers, and functional neuroimaging.

## Materials and Methods

### Participants

Thirteen patients with idiopathic PD confirmed by a neurologist specializing in movement disorders according to the Movement Disorder Society Clinical Diagnostic Criteria (38) accepted to participate in the study. Inclusion criteria were a PD Hoehn and Yahr stage 1–3, age 65 or less, stable dopaminergic oral therapy, negative exercise stress test, and no contraindications to exercise. Exclusions were surgery for PD, cancer, joint diseases, coronary disease, hyperthyroidism, chronic obstructive pulmonary disease, asthma, hypertension, vision defects, history of stroke, anemia, anticoagulant use, pacemaker, insulin pumps, mini-mental score below 24, or the presence of any contraindication for magnetic resonance imaging.

### Study Design

A single-center, parallel-group clinical trial was conducted. Participants were divided in two groups: a control group (CG) and an intervention group (IG) that incorporated high-intensity exercise. Both groups continued with their conventional pharmacological anti-PD medications. Motor function, anthropometric measurements (weight, height, body mass index, waist circumference, percent body fat, percent lean body mass measured by bioelectrical impedance analysis), estimated maximal oxygen consumption (VO_2_max), biomarkers, and functional neuroimaging were assessed before and after complete the intervention in both groups.

The study was conducted at the indoor facilities of the “Vida Activa” wellness center of Fundación Santa Fe University Hospital. VO_2_max and Unified Parkinson's Disease Rating Scale (UPDRS) were measured while individuals were “on” anti-Parkinson's medications. The physicians who took all measurements were blinded to patient group.

### Intervention

The exercise protocol included a conditioning phase of 8 weeks as a progressive adaptation process to exercise. The conditioning phase was intended to allow participants to learn how to ride a tandem bicycle as none of them had used it before, to understand each participant's individual physical needs related to the exercise and to improve the equipment based on individual needs in order to provide greater comfort during exercise and allow some time for its progressive adaptation to the body.

The conditioning phase consisted of session of 30–40 min length, one session per week for the first 4 weeks and two sessions per week for the last 4 weeks. Each session began with 5 min of warmup (low resistance pedaling at 30–40 rpm), followed by 20 min of cycling at 50–60% of their individual's maximum heart rate (HR) (40–60 rpm) and ended with a cool down period of 5 min of cycling at 30–40 rpm. Participants performed 10 min of stretching exercise after each session. Rating of perceived exertion (on the Borg scale) and HR (speed and cadence) were monitored by a general practitioner physician during each session.

We considered the conditioning phase to be very important in order to improve patient engagement, increase patient's confidence and self-efficacy, and build a social network among patients and their families that increases adherence to the intervention and helps prevent exercise-related injury.

After the conditioning phase, patients participated in a high-intensity forced cycling program on a stationary tandem bicycle, three times a week for 16 weeks. Each training session consisted of a 10-min warm-up (low resistance pedaling at 30–40 rpm), followed by 20 min cycling at 80% of each individual's maximum HR while pedaling at 80 rpm or faster and ended with a cool down period of 5 min of cycling at 30–40 rpm. Participants performed 10 min of stretching exercise after each session. To rate perceived exertion (on the Borg scale) and HR, speed and cadence were monitored by a general practitioner during each session. HR was collected with a Polar^TM^ heart rate monitor worn on the chest; pedaling variables were measured using a bicycle speedometer and odometer. Healthy physical educators partnered with each patient to provide motivation and to assist individuals to keep the pace and cadence to reach 80% of their individual's maximum HR during each session. All participants successfully completed each exercise session.

### Clinical Assessment

We measured in all participants at study start and end the UPDRS, estimated maximal oxygen consumption (VO_2_max), and anthropometry.

### Functional Magnetic Resonance Imaging

Subjects were scanned on a General Electric Signa Excite 1.5 T scanner at a University Hospital. T1 images were acquired using 3D magnetization-prepared rapid gradient echo (MP-RAGE) sequence, repetition time (TR) = 13.12 ms, echo time (TE) = 4.2 ms, flip angle = 15°, field of view (FOV) = 240 × 240 mm, voxel size = 1 mm isotropic. Resting and task fMRI data were acquired using gradient-echo echoplanar imaging (EPI) sequence, with TR = 3,000 ms, TE = 60 ms, flip angle = 90°, FOV = 240 × 240 mm, voxel size = 3.75 × 3.75 × 7 mm. Each run of resting fMRI scan lasted 6 min, producing 180 volumes of 3D images. For the hand motor task, subjects were required to tap with the index fingers for periods of 30 s alternating with 30 s of rest.

Data were analyzed using the package SPM12, Department of Imaging Neuroscience Group, London, UK. The images were realigned spatially to the first series of each subject to correct the head movement; slice timing correction ascending (39), and slice 10 was taken as a reference. Functional images were normalized to standard stereotaxic space Atlas Montreal Neurological Institute (MNI). Finally, images were spatially smoothed with a three-dimensional 7-mm full width half maximum isotropic Gaussian kernel filter to improve S/N ratio. For each task we use parametrical analysis of first and second level. The model parameters were canonical hemodynamic response function (HRF) with time and derivatives, six multiple regressors for motion head to reduce intra- and intersubject variability, and high-pass filter of 128.

The second-level analysis consisted in a full factorial design; the first factor was the time of observation (pretreatment and post-treatment), the second factor was the group (IG and CG). We used an alpha of 0.05 without correction for all analyses. The greater cluster with 10 voxels was included. The locations of the statistical findings are reported in a space coordinate (*x, y, z*) developed by the Consortium of Brain Mapping, Montreal Neurological Institute. Imaging data from resting fMRI were preprocessed using Statistical Parametric Mapping 8 (SPM5, http://www.fil.ion.uclac.uk/spm) and Data Processing Assistant for Resting-State fMRI (40, 41). In brief, the preprocessing included slice timing, head movement correction, spatial normalization, band-pass filtering (0.01–0.08 Hz), and global normalization. We decided to exclude the initial 10 volumes as part of the standardized process of the software (FSL, https://fsl.fmrib.ox.ac.uk/fsl/fslwiki/) used for the task block analysis in order to allow magnetization to reach an equilibrium and reduce the noise before reaching the steady-state of imaging; the remaining volumes were then corrected and realigned to the first volume to correct displacement due to head motion (42).

Next, the individual T1-weighted images were co-registered to the mean realigned functional images using a linear transformation; the T1 was segmented into gray matter, white matter, and cerebrospinal fluid tissue maps followed by non-linear normalization into the Montreal Neurological Institute space. Temporal band-pass filtering (0.01–0.08 Hz) was performed on the residual time series of each voxel to reduce the effect of low-frequency drift and high-frequency noise (41). The final step in preprocessing was a spatial smoothing with an isotropic Gaussian kernel of 4 mm FWHM.

The functional connectivity was estimated with a procedure based on seeds or region of interest (ROI). The time sequence is extracted from each seed and that date is used as regressor for a linear correlative analysis. The correlation coefficient among all seeds was calculated, and subsequently, a symmetric and weighted matrix of connectivity for each subject was created. We used a whole brain approach, and the seeds were specified by the parcellation of automatic anatomical labeling or AAL atlas (43).

In order to identify changes in networks among pre- and post-test in the connectivity matrices of functional connectivity, a network-based statistic (NBS) was done. NBS allows to identify any potential connected structures formed by an appropriately chosen set of supra-threshold links, and the topological extent of any such structure is then used to determine its significance (44, 45). The parameters to analysis were 5,000 permutations and *p*-value of 0.05.

Imaging data were preprocessed using SPM8 (http://www.fil.ion.ucl.ac.uk/spm) and DPARSF (http://rfmri.org/DPARSF). Functional connectivity was obtained with the seed method and the atlas AAL. The matrix of correlation of 116 regions of interest was analyzed by network-based statistic (44) (NBS, https://www.nitrc.org/projects/nbs/).

### Biochemical Measurements

Biomarkers were measured with a MILLIPLEX® MAP human neurodegenerative disease panel (HNDG3MAG-36K, Millipore, USA). Antibody-coated, fluorescently labeled magnetic beads were incubated overnight with diluted plasma samples. After addition of a biotinylated detection antibody and extensive washing, streptavidin–phycoerythrin was added and the fluorescence of beads and phycoerythrin captured in a Luminex MAGPIX® (Millipore, USA) apparatus. Data were analyzed with the Xponent® (Austin, TX, USA) software. The lower limit of detection was 2 pg/ml for BDNF, PDGF-AA, and RANTES; 24 pg/ml for soluble intercellular adhesion molecule-1 (ICAM-1), MPO, cathepsin D, PDGFAB/BB, and NCAM; and 61 pg/ml for soluble vascular adhesion molecule-1 (sVCAM-1).

### Ethical Aspects

The study was approved by the Ethics Committee of Fundación Santa Fe de Bogotá, according to minute CCEI-2342 of November 25, 2014. All study patients provided written informed consent. Please also see below the declaration on *Ethics Approval and Consent to Participate*.

### Statistical Analysis

Within-group changes in continuous variables (UPDRS, VO_2_max, anthropometric measures, and biomarker levels) were performed using paired Student's *t*-tests. Between-group comparisons in the change in continuous variables were performed using analysis of covariance (ANCOVA), with baseline values as covariates and intervention group as fixed factor. Changes in continuous variables were correlated using Spearman's correlation coefficient.

## Results

The CG had three male and four female patients. The IG had four male and two female patients. None of the baseline variables differed significantly between groups (Table 1).

**Table 1:** Baseline characteristics of study participants.

	**Control group**	**Intervention group**
Sex M/F	3:4	4:2
Age (years)	56.0	57.8
Time since diagnosis (range in years)	2–24	2–14
Body mass index (kg/m^2^)	26.7	27.5
% body fat	22.8	19.6
% muscle mass	52.1	56.3
Waist circumference (cm)	91.5	94.0
VO_2_max (ml O_2_/kg/min)	18.7	19.4

### Clinical Severity

**Figure 1:**
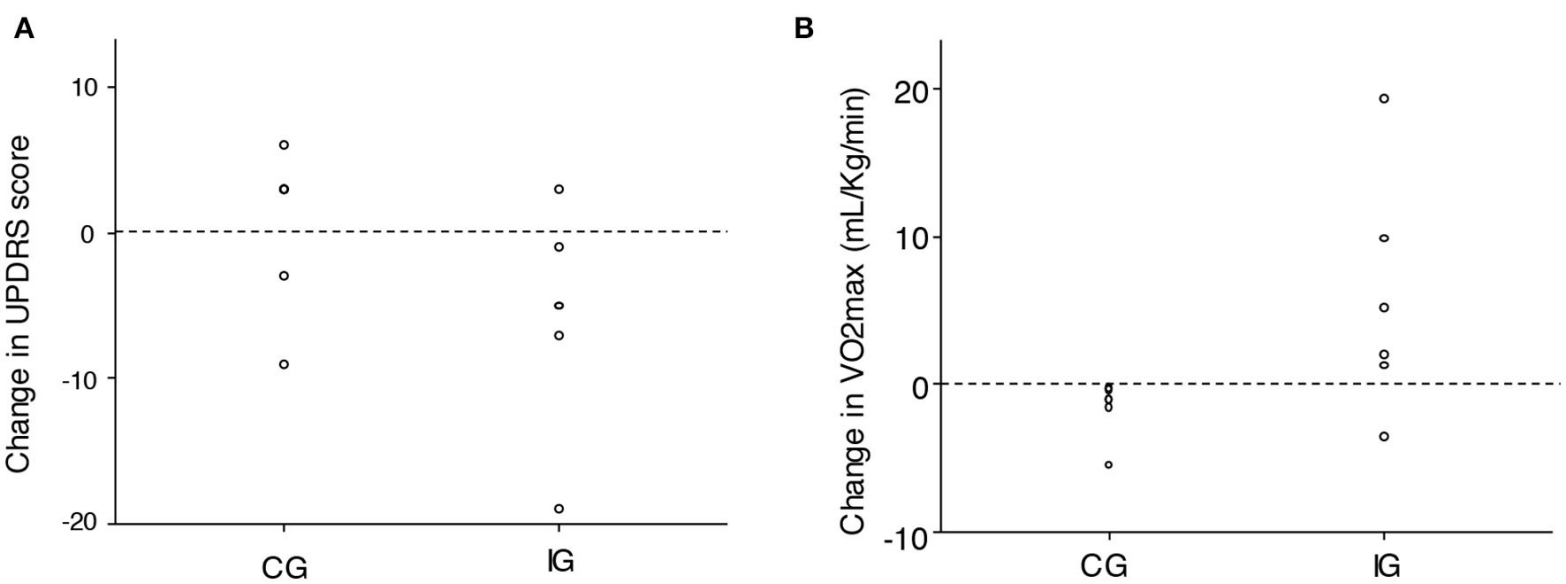
Change in **(A)** total Unified Parkinson's Disease Rating Scale and **(B)** VO_2_max in the study groups. IG, intervention group; CG, control group.

The CG experimented a deterioration in VO_2_max during the study duration (from 18.7 to 17.1 ml/kg/min, intragroup *p* = 0.092), while VO_2_max increased in the IG (from 19.4 to 25.1 ml/kg/min, intragroup *p* = 0.008; Figure 1). The between-group comparison in the change in VO_2_max reached statistical significance (*p* = 0.041). Changes in weight, BMI, percent body fat, percent lean body mass, and waist circumference did not differ between groups (Table 1).

Baseline mean UPDRS was 57.7 in the CG and 54.8 in the IG. Patients in the CG experienced a mean 0.9-point increase (intragroup *p* = 0.67), while those in the IG had a mean 5.7-point decrease (intragroup *p* = 0.12; Figure 1). The *p*-value for the between-group difference was *p* = 0.11.

Mean score in Part I of UPDRS [non-motor aspects of experiences of daily living (nM-EDL)] increased in both the CG (+2.7 points) and IG (+0.3 points) (between-group *p* = 0.12). Part II of UPDRS [motor aspects of experiences of daily living (M-EDL)] remained constant in both groups (+0.1 points in CG, +0.3 points in IG, *p* = 0.96). Part III of UPDRS (motor examination) was reduced by 6.7 points in the IG, while it went down by only 2.1 points in the CG. However, the difference did not reach statistical significance (*p* = 0.32). Mean score in Part IV of UPDRS (motor complications) also remained constant (+0.1 points in the CG, +0.5 points in the IG, *p* = 0.61).

### fMRI

**Figure 2:**
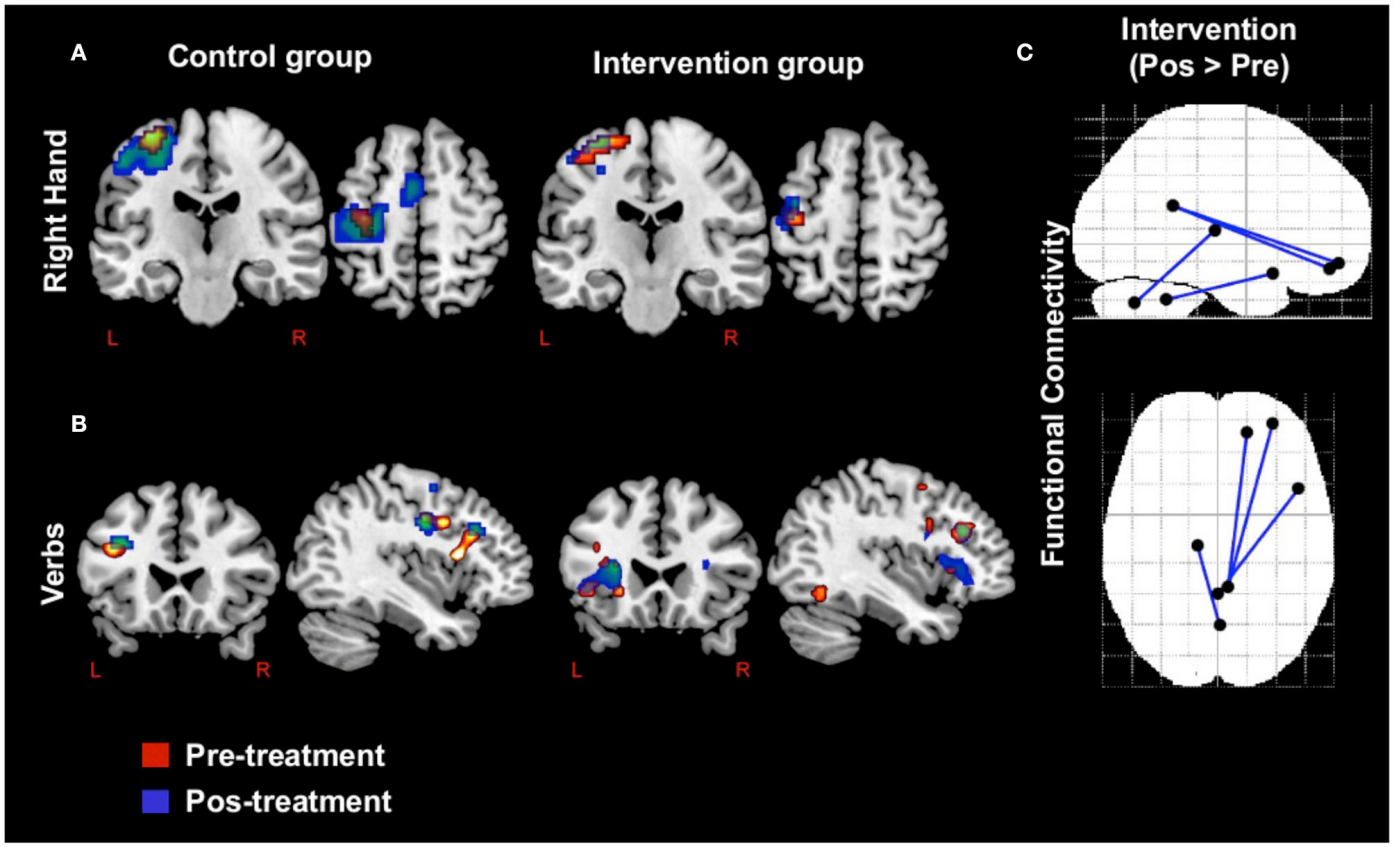
Activations and connectivity in functional magnetic resonance imaging. **(a)** Activations in right hand motor task by group and condition. **(b)** Activations in verb generation task by group and condition. **(c)** Comparison between conditions with network-based strategy in resting functional magnetic resonance imaging.

We found similar baseline activations in both groups in the motor right-hand task, including the left pre-central gyrus and cerebellum. The IG exhibited greater activation of the right fusiform gyrus and decreased activation of the left pre-central gyrus at study end, relative to the CG. In the verb generation task, pretreatment activations were similar in the IG and CG, involving portions of frontal cortex like the left pars triangularis. Final images revealed lower activation of this area in the IG compared to the CG. Network-based strategy (NBS) revealed post-exercise increases in functional connectivity between the right posterior cingulum and the middle frontal and superior orbital gyri, as well as between the vermis and the thalamus and posterior temporal gyrus (Table 2 and Figure 2).

**Table 2:** Functional magnetic resonance imaging main activations by task, group, and condition.

	**Control group**					**Intervention group**				
	**Region**	***t*-value**	***x***	***y***	***z***	**Region**	***t*-value**	***x***	***y***	***z***
**RIGHT HAND TASK**										
Baseline	Left pre-central gyrus	7.0	−36	−19	65	Left pre-central gyrus	6.7	−36	−19	65
	Left cerebellum (crus 1)	4.3	−30	−70	−31	Right fusiform gyrus	6.6	21	−85	−10
	Right cerebellum (VI)	4.1	24	−76	−22	Left cerebellum (crus 1)	5.2	−21	−85	−16
	Left inferior parietal lobule	3.7	−51	−46	53	Left posterior medial frontal gyrus	4.1	−9	−7	53
Post-treatment	Left pre-central gyrus	9.0	−36	−19	65	Right fusiform gyrus	7.2	24	−85	−10
	Left post-central gyrus	6.0	−51	−22	50	Left cerebellum (VI)	5.4	−21	−82	−16
	Left inferior parietal lobule	4.1	−51	−43	53	Left pre-central gyrus	5.2	−36	−19	65
	Left posterior medial frontal gyrus	4.7	−3	−1	59	Left mid-cingulate cortex	4.1	−12	−4	50
						Left cerebellum (VI)	4.0	−33	−67	−19
**VERB GENERATION TASK**										
Baseline	Left fusiform gyrus	4.1	−27	−82	−13	Left inferior frontal gyrus (pars triangularis)	5.3	−39	29	26
	Left inferior frontal gyrus (pars triangularis)	4.0	−39	14	14	Left superior medial gyrus	4.8	−9	17	47
						Left inferior frontal gyrus (pars orbitalis)	4.2	−48	20	−4
						Left middle frontal gyrus	4.5	−36	5	56
						Left posterior medial frontal gyrus	4.2	−6	2	62
Post-treatment	Left posterior medial frontal gyrus	5.9	−6	−1	65	Right fusiform gyrus	4.2	27	−82	−1
	Left inferior frontal gyrus (pars triangularis)	5.6	−36	26	29	Left insula lobe	3.7	−27	20	11
	Left pre-central gyrus	5.3	−39	−7	35	Left inferior frontal gyrus (pars triangularis)	3.5	−39	29	26
	Right fusiform gyrus	4.4	21	−85	−10	Left middle frontal gyrus	3.0	−15	−10	56

### Changes in Biomarkers

In the IG, mean BDNF increased from 27.2 to 218.7 pg/ml (intragroup *p* = 0.002), PDGF-AA from 22.9 to 192 pg/ml (intragroup *p* = 0.038), and PDGF-AB/BB from 16.3 to 366 pg/ml (intragroup *p* = 0.013; Table 3). Contrastingly, no biomarker changed significantly in the CG. Finally, the between-group comparisons of change in BDNF (between-group *p* = 0.005) and RANTES (between-group *p* = 0.030) reached statistical significance.

**Table 3:** Changes in plasma concentrations of biomarkers, by study group.

	**Control group**			**Intervention group**			**Between-groups *p*-value**
	**Baseline**	**Final**	**Intragroup *p*-value**	**Baseline**	**Final**	**Intragroup *p*-value**	
BDNF (pg/ml)	260.0	149.6	0.35	20.63	207.1	0.028	0.005
Cathepsin D (pg/ml)	4,824	3,559	0.92	3,637	3,716	0.50	0.63
sICAM-1 (pg/ml)	1,899	1,546	0.23	2,145	1,546	0.043	0.41
MPO (pg/ml)	28,150	3,559	0.14	46,992	3,733	0.080	0.78
PDGF-AA (pg/ml)	143.3	83.4	0.50	23.8	157.6	0.028	0.13
RANTES (pg/ml)	837.3	1,210	0.18	202	2,850	0.18	0.03
NCAM (pg/ml)	6,387	5,731	0.87	8,152	8,198	0.75	0.43
PDGF-AB/BB (pg/ml)	576.9	399.2	0.74	16.4	336.4	0.028	0.96
sVCAM-1 (pg/ml)	2,916	2,645	0.25	2,968	3,202	0.46	0.36

### Correlation Between Changes in Biomarkers and Changes in Clinical Variables

**Figure 3:**
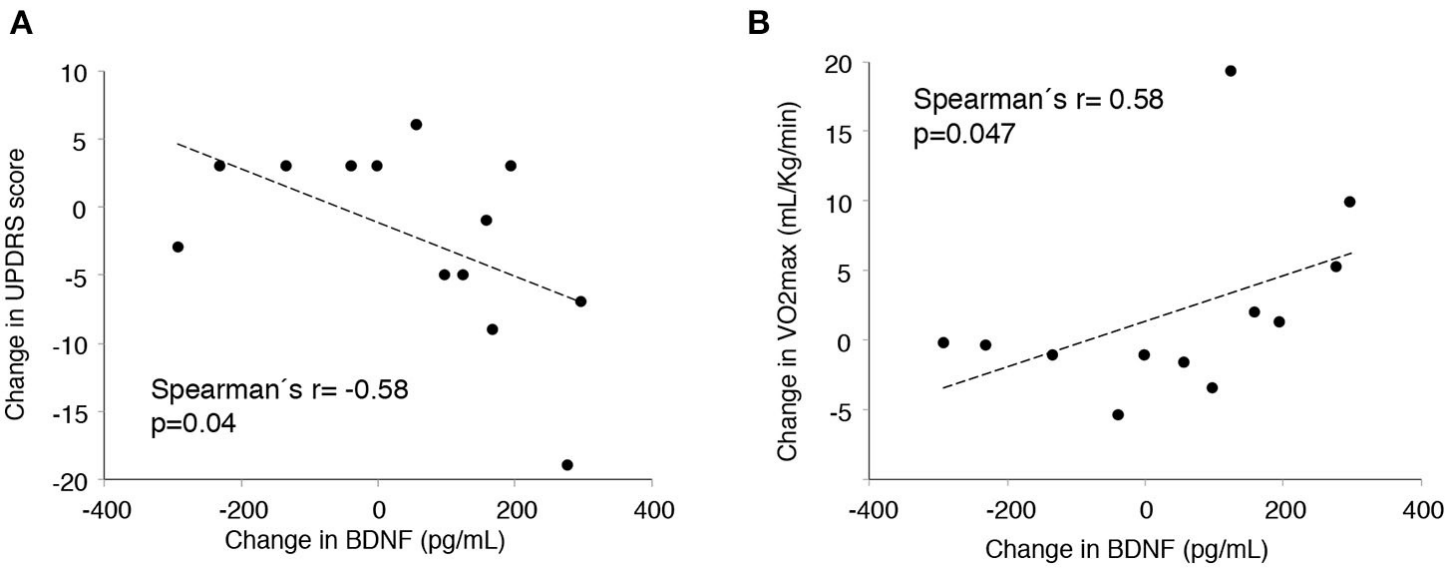
Correlation between change in plasma brain-derived neurotrophic factor (BDNF) and change in clinical variables. **(A)** Unified Parkinson's Disease Rating Scale. **(B)** VO_2_max.

Larger increases in BDNF were associated with greater improvements in UPDRS (correlation between change in plasma BDNF and change in the total UPDRS *r* = −0.58, *p* = 0.040). Changes in BDNF were also positively correlated with improvements in VO_2_max (*r* = 0.58, *p* = 0.047; Figure 3). Changes in NCAM were negatively correlated with changes in percent body fat (*r* = −0.79, *p* = 0.001).

### Adverse Events

We did not encounter any physical or psychological adverse events during the course of the study.

## Discussion

To our knowledge, this pioneer study is the first to integrate clinical variables, fMRI, and biomarkers to assess the impact of a high-intensity tandem bicycle intervention in patients with PD. Despite some results not reaching statistical significance due to limited sample size, findings from this study suggest that high-intensity tandem bicycle induces improvements in clinical, biochemical, and functional neuroimaging variables in PD patients.

VO2 max improved in the IG, but remained constant in the CG, a difference that reached statistical significance. A similar trend was observed for UPDRS, even though in this case, the difference was not statistically significant. In fMRI, exercise promoted activation of the right fusiform gyrus during the motor task and functional connectivity between the cingulum and areas of the frontal cortex, and between the cerebellar vermis and the thalamus and posterior temporal gyrus. Plasma BDNF levels increased more than 10-fold in the IG and decreased in the CG, a significant difference. Larger increases in plasma BDNF correlated with greater decreases in UPDRS. These findings are similar to those from previous studies showing that high-cadence tandem cycling improves motor function and mobility in patients with PD (6).

Several previous clinical studies have documented the positive impacts of exercise on PD (5–9, 19, 20). A systematic review of 104 studies concluded that there is good evidence supporting benefits of exercise on UPDRS (35), especially on the M-EDL subscale and motor examination, but this effect may vary according to exercise modality.

We found significant increases in plasma BDNF and PDGF-BB in the IG. A significant reduction in sICAM-1 was also achieved. Intracerebroventricular administration of PDGF-BB to a mouse model of PD restored striatal dopamine transporter binding sites and expression of nigral tyrosine hydroxylase (27). In addition, the impact of exercise on BDNF has been reported previously. Eight weeks of interval training in stationary bicycle incremented BDNF in PD patients (46, 47), and BDNF has shown potential to improve dopaminergic neuron survival (21, 22). Of note, plasma BDNF appropriately reflects concentrations in central nervous system (48, 49). We found a marked correlation between increases in BDNF and improvements in UPDRS. The negative association between changes in this neurotrophin and changes in the clinical severity of PD is consistent with previous results (46, 47). Thus, BDNF biology, prior studies, and our own results support a potential involvement of BDNF on the impact of exercise on PD. Concerning sICAM-1, this molecule may constitute a marker of sustained brain inflammatory processes in both animals and humans (50). Higher plasma sICAM-1 was observed in stage 1 and 2 patients with PD when compared to healthy controls (51), suggesting a role of inflammatory agents in PD pathogenesis that could be mitigated by forced, high-intensity exercise.

Similar to earlier fMRI studies in PD (52, 53), we found baseline cerebellar hyperactivation in our PD patients. Concerning changes in fMRI induced by exercise, a prior tandem bicycle trial in PD found increased exercise-induced activity in the globus pallidus, thalamus, primary motor, and supplementary motor areas (54). None of these areas appeared to be differentially activated by the intervention in our study. Interestingly, what we did find was an exercise-induced activation of the fusiform gyrus that, to our knowledge, had not been documented previously. The fusiform gyrus is involved in the executive function that is frequently altered in PD patients as may occur in patients with rigidity and bradykinesia. If there is increased cortical activation with exercise, we consider that the executive function may deteriorate more slowly or may even improve in PD patients who exercise, reinforcing the importance of exercising in PD patients, as reductions in the gray matter volume of the fusiform gyrus (along with other temporal areas) seems to be associated with cognitive impairment and poorer executive function in PD patients (33, 55).

A previous study that evaluated an 8-week forced-rate pedaling exercise program reported stronger connectivity between the motor cortex and the ipsilateral thalamus (19). Similarly, we found increased connectivity between thalamus and posterior temporal gyrus in the IG. Hence, our exercise program induced cortical and connectivity changes associated with positive effects on PD.

High-intensity protocols based on tandem bicycle have shown to improve motor function, rigidity, and bradykinesia, as well as induce activity-dependent neuroplasticity (7, 12, 17, 18), probably by promoting high-frequency entry patterns to the sensorimotor cortex. Forcing a high pedaling rate seems to be a determinant of the effects of cycle training in PD (56), probably through induction of increases in afferent stimuli from osteotendinous structures (55). Other studies have proposed different hypothetic explanations regarding the mechanisms that improve motor function in PD patients after cycling.

Cycling may enhance both extrinsic and intrinsic sensory feedbacks from the periphery and the subsequent activation of basal ganglia circuits, which may enhance central motor processing (57); the pedals of a stationary bicycle inherently offer PD patients the mechanical constraint of a constant movement amplitude (57, 58).

Data from our study suggest that exercise may trigger several simultaneous mechanisms that integrated increased brain activation and improved activation of basal ganglia circuits and release of biochemical factors that act as potential neuroprotective and neurotrophic mediator agents (12–20). Our study provides comparative data against other high-intensity cycling interventions.

Additionally, these findings show that individuals with PD are able to participate in a high-intensity cycling intervention and benefit from it. While these findings do not directly answer the question regarding the optimal training variables (intensity/duration/frequency), they contribute to understand the mechanisms that improve motor function in PD patients after cycling.

Further examination of the correlation between changes in neuroimages, biomarkers, and clinical variables of PD induced by longer interventions are needed in order to develop individualized and more specific exercise-training programs.

### Study Limitations

Despite the encouraging results, our study has several limitations. The integration of biomarkers and fMRI to the clinical assessments makes this study not only unique and interesting but also highly expensive and logistically complex. Due to these considerations, we used a small convenience sample of 13 patients. In addition, the design was not randomized due to the requirement of a high degree of collaboration (continued attendance, adherence to exercise routines, multiple complex evaluations) by study participants.

Even so, the findings from our study provide a novel approach and original data to understand the mechanisms that improve motor function in PD patients after cycling.

## Conclusion

Findings from this study suggest that high-intensity tandem bicycle improve motor function and biochemical and functional neuroimaging variables in PD patients. Further research is needed to better understand the mechanisms underlying the improvement of motor function, as well as the type, training variables (intensity/duration/frequency), and dose–response involved in each exercise training practice.

## Data Availability Statement

The datasets generated and/or analyzed during the current study are not publicly available due to institutional policies protecting patient privacy but are available in de-identified from the corresponding author on reasonable request.

## Ethics Statement

The studies involving human participants were reviewed and approved by Fundacion Santa Fe de Bogota. The patients/participants provided their written informed consent to participate in this study.

## Author Contributions

CS and ME participated in study conception, writing the protocol, writing the application for funding, coordinating the administrative task to buy study equipment, patient enrollment, consenting patients, patients evaluation, data collection, data analysis, and manuscript writing. JB participated in study conception, looking for funding, coordinating the administrative task to buy study equipment, patient enrollment, data collection, data analysis, and manuscript editing. CM participated in study conception, writing the protocol, writing the application for funding, coordinating the administrative task to buy study equipment, patient enrollment, consenting patients, data collection (laboratory analyses), data analysis, and manuscript writing. GS and NU participated in patient enrollment and evaluation, consenting patients, data collection (neuroimaging), data analysis (neuroimaging), and manuscript revision. OB-P participated in study conception, data collection (neurology data), data analysis, and manuscript writing revision. GM participated in study conception, search for funding, data analysis, and manuscript revision. LS, EH, MP-J, and AC-M participated in patient enrollment, data collection, and manuscript revision.

## Conflict of Interest

The authors declare that the research was conducted in the absence of any commercial or financial relationships that could be construed as a potential conflict of interest.

## Abbreviations

PD: Parkinson's disease
IG: intervention group
CG: control group
UPDRS: Unified Parkinson's Disease Rating Scale
VO_2_max: maximal oxygen consumption
fMRI: functional magnetic resonance imaging
BDNF: brain-derived neurotrophic factor
L-DOPA: L-3,4 dihydroxyphenylalanine
RANTES: regulated on activation, normal T expressed and secreted
MPO: myeloperoxidase
NCAM: neural cell-adhesion molecule
PDGF: platelet-derived growth factor isotype BB-BB
MP-RAGE: 3D magnetization-prepared rapid gradient echo
DPARSF: data processing assistant for resting-state fMRI
sICAM-1: intercellular adhesion molecule-1
sVCAM-1: soluble vascular adhesion molecule-1
nM-EDL: non-motor aspects of experiences of daily living
M-EDL: motor aspects of experiences of daily living.
